# Emergency department visits and unanticipated readmissions after liver transplantation: A retrospective observational study

**DOI:** 10.1038/s41598-018-22404-8

**Published:** 2018-03-06

**Authors:** Seung-Young Oh, Jeong Moo Lee, Hannah Lee, Chul-Woo Jung, Nam-Joon Yi, Kwang-Woong Lee, Kyung-Suk Suh, Ho Geol Ryu

**Affiliations:** 1 Department of Surgery, Seoul National University College of Medicine, Seoul, South Korea; 2 Department of Anesthesiology, Seoul National University College of Medicine, Seoul, South Korea

## Abstract

Improved survival after LT are likely to result in increased healthcare resource utilization. The pattern and risk factors of emergency department (ED) visits and unanticipated readmissions, associated cost, and predictors of healthcare resource utilization after liver transplantation (LT) patients who received LT between 2011 and 2014 were analyzed. A total of 430 LT recipients were enrolled and the 1 year all-cause mortality was 1.4%. ED visits occurred in 53% (229/430) and unanticipated readmissions occurred at least once in 58.6% (252/430) of the patients. Overall risk factors for ED visits after LT included emergency operation [OR 1.56 (95%CI 1.02–2.37), *p* = 0.038] and warm ischemic time of >15 minutes [OR 2.36 (95%CI 1.25–4.47), *p* = 0.015]. Risk factors for readmissions after LT included greater estimated blood loss during LT [OR 1.09 (95%CI 1.02–1.17), p = 0.012], warm ischemic time of >15 minutes [OR 1.98 (95%CI 1.04–3.78), *p* = 0.038], and hospital length of stay of >2 weeks.

## Introduction

Liver transplantation (LT) is one of the most expensive medical procedures which requires an extensive preoperative evaluation and meticulous postoperative management^1^. Improved long term post-transplant survival and the potential for infectious and noninfectious complications after LT calls for significant healthcare resources, posing a financial burden on the healthcare system and the patients^2^.

From the patient’s perspective, there is little information regarding what to expect after discharge from a successful liver transplantation. Unexpected hospital visits after LT, especially emergency department (ED) visits and hospital readmissions, can be disturbing and disruptive. It can also be financially challenging as costs of medications for immunosuppression are already high. However, little is known about the frequency and patterns of health care resource utilization after LT.

The aim of our single center retrospective observational study was to display the pattern of ED visits and readmissions after LT and the associated costs. Predictors of healthcare resource utilization after LT were also evaluated.

## Results

### Patient characteristics

A total 442 patients were identified and after excluding 10 patients who died during the admission of receiving LT and 2 patients with no history of SNUH outpatient department visit after discharge, 430 patients were included in the analysis. As described in Table 1, 70.5% (303/430) were living donor LTs and the overall average MELD score was 16.9. The LOS after LT was 20.5 ± 17.8 days. One year all-cause mortality after discharge was 1.4% (6/430).

**Table 1:** Patient characteristics.

Characteristics	n = 430
Age (years)	53.8 ± 9.8
Male/Female	297 (69.1)/133 (30.9)
Body mass index (kg/m^2^)	23.9 ± 3.6
Coexisting liver disease	
Hepatitis B virus	269 (62.6)
Hepatitis C virus	47 (10.9)
Hepatocellular carcinoma	238 (55.4)
Alcoholic liver cirrhosis	73 (17.0)
Others	26 (9.9)
Model for End-stage Liver Disease score	16.9 ± 9.1
Comorbidities	
Hypertension	72 (16.7)
Diabetes mellitus	100 (23.3)
Coronary heart disease	7 (1.6)
Chronic kidney disease	5 (1.2)
Cerebrovascular disease	5 (1.2)
Donor age (years)	37.1 ± 13.1
Donor Sex,	
Male/Female	279 (65.3)/148 (34.7)
Living donor/deceased donor	303 (70.5)/127 (29.5)
Elective/emergency operation	296 (68.8)/134 (31.2)
Graft recipient weight ratio	1.3 ± 0.4
Cold/warm ischemic time (minutes)	69.9 ± 71.8/34.9 ± 36.3
Estimated blood loss (mL)	3305 ± 4687
ABO mismatch	15 (3.5)
Postoperative hospital length of stay (days)	20.5 ± 17.8

Data are presented as mean ± SD, or number (%).

### ED visits after LT

**Figure 1:**
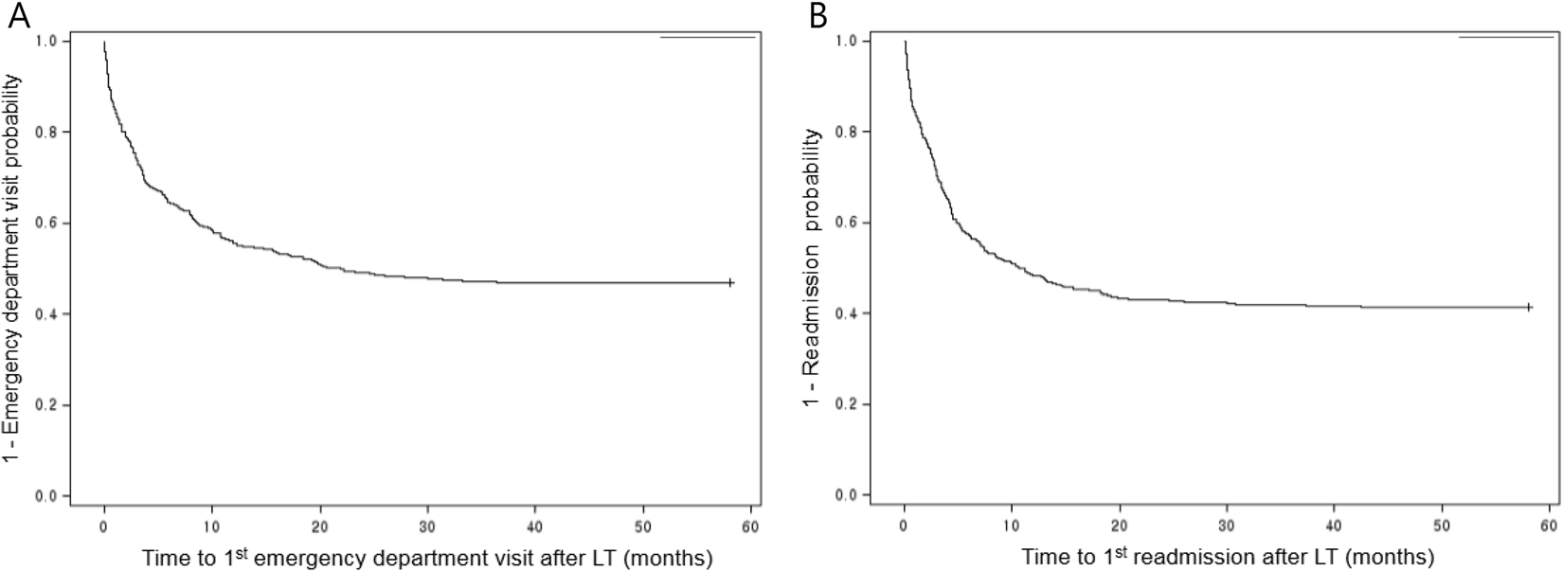
Time to 1^st^ emergency department visit and readmission after liver transplantation.

There were a total of 516 ED visits after discharge for the 430 patients during an average follow-up period of about 30 months (926.3 ± 382.45 days). ED visits occurred at least once in 53% (229/430) of the 430 patients. The probability of ED visit increased from 15.3% (66/430) at 30 days to 43.6% (169/388) at 1 year (Fig. 1A, Table 2). The number of patients who visited the ED twice (118) were about half of those who visited ED only once (229). The number of patients who visited the ED 3 times (65) were about half of those who visited the ED twice. The average time to first ED visit after discharge from LT was 188.4 ± 235.0 days (Table 3). Overall, 45.7% (236/516) of ED visits led to readmissions.

**Table 2:** Healthcare resource utilization after discharge from liver transplantation.

	Time after discharge from liver transplantation					
	≤7 days (n = 430)	≤30 days (n = 430)	≤6 months (n = 419)	≤1 year (n = 388)	≤2 years (n = 286)	≤3 years (n = 180)
ED visits						
0	405 (94.2)	364 (84.7)	271 (64.7)	219 (56.4)	147 (51.4)	88 (48.9)
1	22 (5.1)	52 (12.1)	92 (22.0)	93 (24.0)	66 (23.1)	44 (24.4)
2	2 (0.5)	12 (2.8)	29 (6.9)	45 (11.6)	35 (12.2)	18 (10.0)
3	1 (0.2)	2 (0.5)	20 (4.8)	16 (4.1)	20 (7.0)	14 (7.8)
4			3 (0.7)	6 (1.6)	6 (2.1)	4 (2.2)
≥5			4 (1.0)	9 (2.3)	12 (4.2)	12 (6.7)
Readmissions						
0	409 (95.1)	361 (84.0)	242 (57.8)	198 (51.0)	136 (47.6)	82 (45.6)
1	21 (4.9)	65 (15.1)	107 (25.5)	88 (22.7)	58 (20.3)	38 (21.1)
2		4 (0.9)	44 (10.5)	54 (13.9)	40 (14.0)	18 (10.0)
3			15 (3.6)	18 (4.6)	13 (4.6)	13 (7.2)
4			9 (2.2)	11 (2.8)	16 (5.6)	9 (5.0)
≥5			2 (0.5)	19 (4.9)	23 (8.0)	20 (11.1)

Data are presented as number (%).

ED, emergency department.

**Table 3:** Time to emergency department visit and readmission after discharge from liver transplantation.

	n	Time after discharge from liver transplantation (days)	Interval from previous visit (days)
ED visit			
1^st^ visit	229	188.4 ± 235.0	
2^nd^ visit	118	284.7 ± 284.1	157.8 ± 199.2
3^rd^ visit	65	372.5 ± 310.1	180.6 ± 238.1
4^th^ visit	36	450.2 ± 341.2	137.6 ± 160.6
5^th^ visit	25	496.0 ± 346.5	145.4 ± 263.7
Readmission			
1^st^ readmission	252	157.1 ± 199.6	
2^nd^ readmission	150	259.1 ± 225.2	135.5 ± 172.7
3^rd^ readmission	99	392.4 ± 287.0	133.0 ± 153.0
4^th^ readmission	71	477.7 ± 328.4	127.5 ± 166.1
5^th^ readmission	44	530.2 ± 335.3	115.7 ± 156.2

Data are presented as mean ± SD.

ED, emergency department.

Identified risk factors for ED visits after LT were emergency operation [OR 1.56 (95%CI 1.02–2.37), *p* = 0.038] and warm ischemic time of 15 minutes or longer [OR 2.36 (95%CI 1.25–4.47), *p* = 0.015] (Table 4). Multivariable analysis for risk factors for ED visits broken down by periods after LT include emergency operation and prolonged LOS for the first 30 days after discharge, higher MELD scores for 1–3 months after discharge, and previous ED visits beyond the first 30 days after discharge (Table 4).

**Table 4:** Risk factors for emergency department visit after discharge from liver transplantation.

	Multivariable analysis		
	Risk factor	OR (95% CI)	*p*-value
Emergency department visit after discharge	Operation type, elective	—	
	emergency	1.56 (1.02–2.37)	0.038
	Warm ischemic time (minutes), <15	—	
	≥15	2.36 (1.25–4.47)	0.015
Emergency department visit within 30 days	Operation type, elective	—	
	emergency	2.05 (1.19–3.53)	0.010
	Postoperative hospital length of stay (days)		0.028
	<14	—	
	14≤ and <29	2.42 (1.24–4.73)	0.010
	29≤	2.41 (1.03–5.63)	0.043
Emergency department visit in 31–90 days	Emergency department visit in 30 days	2.92 (1.57–5.42)	0.001
	Model for End-stage Liver Disease score		0.013
	<15	—	
	15≤ and <25	2.48 (1.35–4.58)	0.004
	25≤	1.92 (0.93–3.97)	0.077
Emergency department visit in 91–180 days	ED Emergency department visit in 31–90 days	3.61 (2.00–6.52)	<0.001
Emergency department visit in 181–365 days	Emergency department visit in 91–180 days	2.66 (1.50–4.72)	0.001
Emergency department visit after 1 year	Emergency department visit in 181–365 days	3.05 (1.81–5.16)	<0.001

### Readmissions after LT

There were a total of 714 readmissions after discharge for the 430 patients during an average follow-up period of about 30 months (926.3 ± 382.5 days). Readmissions occurred at least once in 58.6% (252/430) of the 430 patients. The probability of the first readmissions increased from 16.0% (69/430) at 30 days to 49.0% (190/388) at 1 year (Fig. 1B, Table 2). The average time to first readmission after discharge from LT was 157.1 ± 199.6 days (Table 3). The number of patients with 2 readmissions (150) were about 60% of the number of patients with 1 readmission (252) and the rate of decrease in the number of patients who were admitted more than twice were also approximately 60% (Table 3). Of the 714 readmissions, 33.1% (236/714) were readmitted from the ED.

Identified risk factors for readmissions after LT were donor age less than 60 years [OR 3.65 (95%CI 1.47–9.06), *p* = 0.005], warm ischemic time of 15 minutes or longer [OR 1.98 (95%CI 1.04–3.78), *p* = 0.038], greater estimated blood loss [OR 1.09 (95%CI 1.02–1.17), p = 0.012], and longer postoperative hospital LOS for LT (Table 5). Multivariable analysis for risk factors for readmissions broken down by periods after LT include emergency operation for the first 30 days after discharge; prolonged postoperative hospital LOS and previous readmissions (within 30 days after discharge) for 1–3 months after discharge; previous readmissions for 3–12 months after discharge; and higher MELD scores, coronary artery disease, and previous readmissions for >1 year after discharge (Table 5).

**Table 5:** Risk factors for readmission after discharge from liver transplantation.

	Multivariable analysis		
	Risk factor	OR (95% CI)	*p*-value
Readmission after discharge	Donor age, ≥60	—	
	<60	3.65 (1.47–9.06)	0.005
	Warm ischemic time (minutes), <15	—	
	≥15	1.98 (1.04–3.78)	0.038
	Estimated blood loss	1.09 (1.02–1.17)	0.012
	Postoperative hospital length of stay (days)		0.004
	<14	—	
	14≤ and <29	1.72 (1.12–2.66)	0.014
	29≤	2.89 (1.44–5.79)	0.003
Readmission within 30 days	Coexistence of hepatitis B virus	0.43 (0.25–0.73)	0.002
	Operation type, elective	—	
	emergency	2.30 (1.35–3.92)	0.002
Readmission in 31–90 days	Postoperative hospital length of stay (days)		0.001
	<14	—	
	14≤ and <29	1.63 (0.89–3.00)	0.115
	29≤	4.07 (1.98–8.35)	0.001
	Readmission within 30 days	2.39 (1.32–4.32)	0.004
Readmission in 91–180 days	Estimated blood loss (L)		0.039
	<1	—	
	1≤ and <1.8	0.67 (0.33–1.34)	0.256
	1.8≤ and <3.8	1.43 (0.76–2.72)	0.269
	3.8≤	0.64 (0.32–1.30)	0.218
	Readmission within 31–90 days	2.79 (1.62–4.79)	<0.001
Readmission in 181–365 days	Readmission within 91–180 days	3.15 (1.95–5.10)	<0.001
Readmission after 1 year	Model for End-stage Liver Disease score	1.03 (1.01–1.06)	0.013
	Coronary heart disease	9.34 (1.73–50.41)	0.009
	Readmission within 181–365 days	3.39 (2.10–5.48)	<0.001

### Causes and costs of readmissions

The most common cause of readmission was abnormal values of liver function test (LFT) which accounted for 32.2% of readmissions (230/714) followed by fever (121/714) and abdominal pain (27/714) (Table 6). Abnormal LFT, fever, and unclassified causes (others) accounted for more than 70% of readmissions, regardless of the number of readmissions (Table 7). The most common component of ‘others’ was management for cancer recurrence (96/217) including chemotherapy, trans-arterial chemoembolization, and surgery.

**Table 6:** Hospital length of stay, costs, and multiple readmissions according to causes of readmission.

	n	Hospital LOS (days)	Hospital costs ($)	Multiple readmissions (n, %)
Total	714	8.1 ± 9.9	5485 ± 11229	
LFT abnormality	230	8.0 ± 9.3	4768 ± 7570	60/117 (51.3)
Others	217	5.2 ± 5.3	3819 ± 3838	48/99 (48.5)
Fever	121	11.7 ± 13.0	8524 ± 22149	27/70 (38.6)
Abdominal pain	27	9.5 ± 12.2	6258 ± 8655	3/24 (12.5)
Pruritus	19	4.4 ± 3.1	2857 ± 1819	5/12 (41.7)
General weakness	17	16.1 ± 17.2	10607 ± 16438	2/12 (16.7)
Diarrhea	15	7.1 ± 11.8	4598 ± 8994	
Loss of consciousness	9	9.8 ± 11.3	6598 ± 6166	
Wound problem	9	7.7 ± 7.1	4229 ± 3548	1/8 (12.5)
Ascites	8	22.0 ± 17.7	15397 ± 16049	2/6 (33.3)
Dizziness	7	4.9 ± 3.6	4890 ± 4331	
Dyspnea	7	12.3 ± 11.2	7127 ± 6082	
Jaundice	6	14.3 ± 10.1	7967 ± 4431	
Hematochezia	5	6.2 ± 5.6	3799 ± 1968	
Chest pain	4	8.0 ± 13.3	10601 ± 14784	1/3 (33.3)
Tube problem	3	5.0 ± 6.7	2534 ± 2533	
Edema	2	12.5 ± 16.3	9643 ± 9750	
Hematemesis	2	11.0 ± 12.7	18385 ± 16945	
Hematuria	2	1.5 ± 0.7	822 ± 588	1/1 (100.0)
Vomiting	2	2.5 ± 2.1	1974 ± 125	

Data are presented as mean ± SD, and number with percentage.

LOS, length of stay; LFT, liver function test.

**Table 7:** Hospital length of stay, costs, and causes of readmission according to number of readmissions.

	1^st^ readmission (n = 252)	2^nd^ readmission (n = 150)	3^rd^ readmission (n = 99)	4^th^ readmission (n = 71)	5^th^ readmission (n = 44)
Hospital LOS (days)	8.9 ± 11.4	7.8 ± 8.9	7.5 ± 8.9	6.3 ± 6.6	7.8 ± 8.9
Cost ($)	6741 ± 15886	4791 ± 5677	4965 ± 7976	3706 ± 3349	4557 ± 5542
Cause of readmission (n, %)					
	Abnormal LFT (86, 34.1)	Abnormal LFT (55, 36.7)	Abnormal LFT (36, 36.4)	Others (23, 32.4)	Others (17, 38.6)
	Others (67,26.6)	Others (43, 28.7)	Others (32, 32.3)	Abnormal LFT (19, 26.8)	Abnormal LFT (12, 27.3)
	Fever (28,11.1)	Fever (27, 18.0)	Fever (20, 20.2)	Fever (16, 22.5)	Fever (9, 20.5)
	Abdominal pain (18, 7.1)	Abdominal pain (5, 3.3)	Chest pain (2, 2.0)	Pruritus (3, 4.2)	LOC (2, 4.5)
	Diarrhea (11, 4.4)	Pruritus (4, 2.7)	General weakness (2, 2.0)	General weakness (2, 2.8)	Diarrhea (1, 2.3)
	Ascites (6, 2.4)	Wound problem (4, 2.7)	Hematochezia (2, 2.0)	Chest pain (1, 1.4)	Dizziness (1, 2.3)
	Dyspnea (5, 2.0)	Jaundice (3, 2.0)	LOC (2, 2.0)	Diarrhea (1, 1.4)	General weakness (1, 2.3)
	General weakness (5, 2.0)	Ascites (2, 1.3)	Abdominal pain (1, 1.0)	Dyspnea (1, 1.4)	Hematemesis (1, 2.3)
	Wound problem (5, 2.0)	Dizziness (2, 1.3)	Diarrhea (1, 1.0)	Hematemesis (1, 1.4)	
	Pruritus (4, 1.6)	Diarrhea (1, 0.7)	Edema (1, 1.0)	Hematochezia (1, 1.4)	
	Dizziness (3, 1.2)	Dyspnea (1, 0.7)		LOC (1, 1.4)	
	Jaundice (3, 1.2)	General weakness (1, 0.7)		Tube problem (1, 1.4)	
	LOC (3, 1.2)	Hematuria (1, 0.7)		Vomiting (1, 1.4)	
	Tube problem (3, 1.2)	LOC (1, 0.7)			
	Chest pain (1, 0.4)				
	Edema (1, 0.4)				
	Hematochezia (1, 0.4)				
	Hematuria (1, 0.4)				
	Vomiting (1, 0.4)				

LOS, length of stay; LFT liver function test; LOC loss of consciousness.

**Figure 2:**
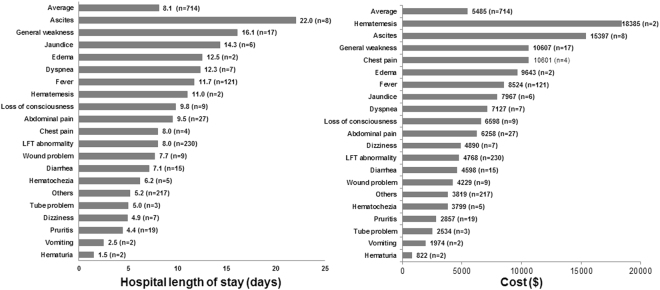
Hospital length of stay (**A**) and hospital costs (**B**) according to the cause of readmission after liver transplantation.

The average LOS for readmissions was 8.1 ± 9.9 days. Readmission due to ascites (22.0 ± 17.7 days) showed the longest LOS, followed by general weakness, and jaundice (Fig. 2A). The average cost associated with readmission was $5485 ± 11229. Readmission due to hematemesis was the most expensive ($18385), followed by ascites, and general weakness (Fig. 2B).

### Risk factors for mortality

There was no mortality within 30 days after discharge from LT and all-cause mortality was 0.2% (1/430) within 3 months after LT, 0.9% (4/430) within 6 months after LT, and 1.4% (6/430) within 1 year after LT. There was no mortality in patients who survived beyond 2 years after LT. Identified risk factors for mortality after LT were coexistence of HCV [OR 3.98 (95%CI 1.15–13.78), *p* = 0.03] and diabetes [OR 3.49 (95%CI 1.09–11.60), *p* = 0.04].

## Discussion

Our study shows that significant healthcare resources are utilized after discharge from LT. ED visits and readmissions occurred at least once in 53% (229/430) and 58.6% (252/430) of the 430 patients. The probability of ED visit and readmission increased from 15.3% and 16.0% at 30 days to 43.6% and 49% at 1 year, respectively.

High MELD scores, case volume of the center, coexistence of HCV, and living donor LT have been reported as risk factors for readmission after LT^3–7^. In contrast to a previous study that reported higher readmission rates and longer hospital LOS in living donor LT patients compared to deceased donor LT patients^4^, living donor LT was not found to be a risk factor of readmission in our study. To the contrary, emergency LT was found to be a risk factor for readmission within 30 days after discharge from LT. Potential causes may include lower MELD scores of living donor LT patients in our study (14.3 vs. 15.2) and the greater amount of experience of our institution (303 cases in less than 3 years vs. 384 cases in more than 5 years among 9 centers). The relatively lower rate of complications after LT in our institution may be contributed to a few factors^8^. LTs using the left liver lobe and pre-existing hepatitis C are known as risk factors for long-term graft failure^9,10^. The most common indication for living donor LT in our institution was hepatitis B with most of these patients receiving LTs using the right liver lobe. In addition, our institution is a large volume center performing more than 150 LTs per year.

Some of the identified risk factors of readmission and ED visit after LT were unexpected. Donor age of less than 60 years was found to be a risk factor for readmission. Although we do not have a plausible explanation, we believe that there may have been a tendency to proceed with deceased donor LT when the donor was relatively young, even when the liver graft was marginal. Warm ischemic time of 15 minutes or longer was also found to be associated with readmission and ED visits. Prolonged warm ischemic time increases hepatic ischemia and reperfusion injury and is related to postoperative complications which can be a cause of frequent readmission. In our study, the average hospital LOS in patients with a warm ischemic time less than 15 minutes was 15.6 days, which was shorter than the overall average LOS. Shorter hospital LOS may reflect less immediate postoperative complications. In addition, warm ischemic time may be influenced by the degree of surgical technique and vascular anatomical variation requiring additional anastomosis. Similar to the relationship between case volume and operative mortality^11^, there may be a relationship between the level of surgical expertise and the risk of readmission and ED visit.

Although no specific type of complication has been identified as a risk factor for readmission^5^, there were specific conditions which accounted for a relatively high proportion of readmissions and repeated readmission in our study. Abnormal LFT was the main cause of readmission in 32.2% of patients, 51.3% of whom were readmitted thereafter at least once more for the same reason. Fever accounted for 16.9% of readmission cause and repeated readmission rate due to fever was 38.6%. Considering these results, we believe that abnormal LFTs and fever are common causes that lead to readmission after discharge and therefore should be managed accordingly.

LT has been reported to reduce hospitalizations by up to 70% except for patients who showed very low preoperative MELD score^3^. Even in patients with a preoperative MELD score ≤9, the LOS was shorter in patients who received LT compared to those who did not. A recent study showed that a protocol composed of organized multidisciplinary clinics, efficient education, and intermediate step between readmission and outpatient clinic reduced the incidence of readmission after LT^12^. Education before discharge was shown to decrease readmission after hematopoietic stem cell transplantation^13^. These studies suggest that there may be opportunities for reducing healthcare resource utilization after LT, which in most cases can be interpreted as better patient outcome. However, readmission rates after LT remain high. Reported 30-day readmission rates after LT range from 26% to 50% and 1 year readmission rates of up to 70%^5,6,14,15^. Another study showed that of the LT recipients who were readmitted within 30 days after discharge half of them required readmission within 7 days after discharge^7^. Our readmission rates of 16% at one month and 49% at one year is lower than the reported rates. The predominant proportion of living donor LT and the low preoperative MELD scores are likely to be determining factors may. Also, as several studies showed that readmission was associated with worse long-term outcome, our low readmission rate seems to be in alignment with our low mortality rate^6,7^.

Improved postoperative survival and shorter hospital LOS have been associated with increased postoperative complication rates^16^. Greater burden of healthcare resource utilization have been shown in LT patients with complications compared to those without complications^17^. Considering LT itself, it is still one of the most resource intense procedures despite significant improvements in procedures and protocols. The estimated cost of LT is $519,300 and the total cost for readmissions within 180 days after LT is $126,900^18^. Another study reported that readmission within 90 days after LT accounted for an additional cost of $43,785 compared to LT recipients who did not require readmission during the same period^7^. Considering that the reported cost of LT in Korea ranges between $50,000 and $60,000, the average hospital cost of $4,199 for readmission after LT in our study seems to be comparable to previous reports.

From the patient’s perspective, information regarding what to expect after LT is relatively scarce. While other studies mostly assessed mortality or complications, the focus of our study was to present a comprehensive pattern of healthcare resource utilization, readmission and ED visits. Similar to previous reports, most of the readmissions and ED visits occurred in the first year after LT, especially in the first month. Although the frequency of complications differ depending on the time frame after LT, infections and graft rejections are more frequent in the first year after LT, whereas cardiovascular disease and malignancies tend to occur at a later stage^19^. Unexpected postoperative events are very concerning to patients and families and we believe that our data may help patients and families understand what to expect in the months and years after LT in a practical sense, in terms of probability of requiring a visit to the ED or readmission, how long the readmission is likely to last, and how much it will cost.

Although this is a retrospective study, we believe that our data is consistent and reliable. All LTs were performed by 3 surgeons, who perform up to 150 LTs per year for the past 10 years as a team with help from a dedicated LT anesthesia team. Adding to the consistency of care, a multidisciplinary ICU team managed the patients postoperatively. Also, the vast majority of LTs in Korea are performed in a few large institutions, including ours. The volume of LTs and the experience may also explain the relatively low rate of readmission rate^20^. Patients were informed of specific conditions that require an immediate visit to the SNUH ED or the outpatient clinic. Patients prefer to be treated at the institution where they received their LT, since only a few institutions have experienced teams that are capable of managing complications after LT.

There are several limitations in our study. Despite the relatively large number of enrolled patients, the nature of this study is retrospective and the inherent limitations of retrospective studies obviously applies. However, considering the consistency of the provided care and the uniformity of data collection (no change in electronic medical records system during the study period), we believe that the quality of the data is reliable. Second, most patients were living donor LT recipients, which is different from most centers in the US and European countries. As reported complications of living donor LT and deceased donor LT are usually different, our results should be interpreted with the different case mix in mind^4^. Also, due to the high proportion of living donor LT, the MELD scores of the LT patients were relatively low which may affect ED visits and readmissions after LT. Third, healthcare resource utilization, especially the ED visits, are very likely to be underestimated as patients may visit EDs of nearby institutions. The number of centers capable of managing complications of LT are very limited. Also, patients have a strong tendency to return to the center that they received their liver transplantation, since previous medical records are not readily available in other centers. Despite being the second largest center in volume of LT in Korea and located in the center of the capitol, patients who have received their liver transplantation at another center are difficult to encounter.

In conclusion, the 30-day and 1-year ED visit rates were 15.3% and 44.4% and the 30-day and 1-year readmission rates were 16.0% and 51.6% after discharge from LT. Patients who did not require readmissions or ED visits in the first 20 months after discharge from LT almost never required unplanned healthcare resources thereafter. Our results may provide practical aspects of life after liver transplantation to patients and their families.

## Methods

This study was a retrospective observational study and the study protocol was approved by the institutional review board of the Seoul National University Hospital (SNUH-1412-099-634). Informed consent was waived by the IRB due to the retrospective study design. All methods employed in this study were performed in accordance with the relevant guidelines and regulations.

### Patients

Adult patients (age ≥ 18 years) who underwent LT at Seoul National University Hospital (SNUH) between March 2011 and December 2014 were included. Patients who died in the hospital after LT before discharge were excluded. Patients were followed up for up to 4 years. All the liver grafts for living donor LTs were procured from SNUH and the liver grafts for deceased donor LTs were procured from Ajou University Hospital, Asan Medical Center, Bundang Cha Hospital, Cheju Halla General Hospital, Chonbuk National University Hospital, Chonnam National University Hospital, Chosun University Hospital, Chungbuk National University Hospital, Chungnam National University Hospital, Daegu Catholic University Medical Center, Dankook University Hospital, Dong-A University Hospital, Dongguk University Medical Center, Eulji University Hospital, Ewha Womans University Mokdong Hospital, Gachon University Gil Medical Center, Gyeongsang National University Hospital, Hallym University Medical Center, Inha University Hospital, Inje University Paik Hospital, Keimyung University Dongsan Medical Center, Konkuk Universtiy Medical Center, Konyang University Hospital, Korea University Medical Center, Kyunghee University Hospital at Gangdong, Kyungpook National University Hospital, Myongii Hospital, National Medical Center, Pusan National University Hospital, Samsung Medical Center, Seoul National University Bundang Hospital, Seoul National University Hospital, Severance Hospital, Soon Chun Hyang University Hospital, St. Carollo General Hospital, The Catholic University St. Mary’s Hospital, Ulsan University Hospital, Wonju Sevrance Christian Hospital, Wonkwang University Hospital, Yeungnam University Medical Center. No organs were procured from prisoners.

### Data collection

Data were collected through review of electronic medical records. To evaluate risk factors for ED visits and readmissions after LT, recipient related factors, donor related factors, and surgery related factors were collected. Recipient related factors included age, sex, body mass index (BMI), type of liver disease, Model for End-stage Liver Disease (MELD) score, underlying comorbidities. Donor related factors included age, sex, and type of donor (living or deceased). Surgery related factors included operation type (elective or emergency), type of graft (whole liver, right lobe, left lobe, or others), graft-recipient weight ratio (GRWR), cold and warm ischemic time (between hepatic vein anastomosis and reperfusion), estimated blood loss, and ABO compatibility between the recipient and the donor. The frequency of missing data was less than 5% except for GRWR (49.3%), cold ischemic time (38.8%), and warm ischemic time (9.3%). Missing data were handled by exclusion from analysis.

### Liver transplantation protocol

All LT surgeries were performed by 3 surgeons who collectively perform more than 150 LTs per year and apply the similar surgical techniques even in detailed procedures. A dedicated LT anesthesia team performed the anesthesia for all LTs during the study period using the same anesthesia protocol^21^.

All donor liver grafts were prepared with the histidine-tryptophan-ketoglutarate solution. End-to-end anastomosis of the hepatic artery and duct-to-duct anastomosis of bile duct were done in succession. Electrolytes and arterial blood gas were monitored and corrected accordingly throughout surgery. All liver recipients were transported to the intensive care unit for postoperative care after surgery.

All liver recipients had received 20 mg of intravenous basiliximab (interleukin-2 receptor antagonist) two hours before LT and postoperative day 4. Immunosuppression after LT was based on triple immunosuppressive regimen with calcineurin inhibitor, mycophenolate mofetil, and steroids. Doses of tacrolimus or cyclosporine were adjusted depending on each patient’s clinical condition and target trough levels are approximately 8–12 ng/mL and 200–300 ng/mL for the first month after LT, followed by 5–8 ng/mL and 100–200 ng/mL thereafter. Mycophenolate mofetil was adjusted according to the associated side effects. Intravenous methylprednisolone 500 mg was given intra-operatively before portal perfusion. It was tapered from 200 mg to 20 mg within 6 days and oral prednisolone was continued at 20 mg daily thereafter. It was tapered to 0–5 mg/day until 6 months post-LT.

Relevant laboratory tests were performed daily during hospitalization after LT and abdominal computed tomography was performed 1 week after surgery to check for hidden abnormalities. Patients with an uneventful hospital course were discharged once the immunosuppressant level was stable, usually at 2 weeks after surgery.

Before discharge from the hospital, LT recipients and their caregivers are strongly encouraged to attend one of the weekly 90-minute education program regarding life after transplantation. The contents consisted of guidelines of daily life after transplantation such as exercise, sexual life and pregnancy, medications including immunosuppressants, diet, and nutrition. Patients were provided with a thermometer, scale, blood pressure monitor, and blood glucose monitor before discharge, and were instructed to check their blood pressure and blood glucose daily. Also, patients were informed of specific conditions that require an immediate visit to the SNUH ED or the outpatient clinic such as fever (>38 °C), severe headache or muscle pain, abdominal pain, vomiting, diarrhea, hematochezia, and rapid weight change. Patients were also provided with a 24 hour accessible phone number for help and instructions in case of urgent situations. Outpatient clinics for liver transplantation patients are accessible every day during regular hours and were referred to the ED after regular hours.

After discharge, LT recipients visited the outpatient clinic weekly for 4 weeks, biweekly for 4 weeks, monthly for 8 weeks, and bimonthly until 1 year after LT. Complete blood count, liver panel, renal panel, coagulation panel, and immunosuppressant level were checked at each outpatient clinic visit. Patients were referred to the ED or readmitted for further evaluation or management when necessary.

### Study outcomes

Frequency and probabilities over time of ED visits, and unanticipated readmissions were evaluated. For each readmission after LT, the initial cause, hospital costs, and hospital length of stay (LOS) were assessed. The risk factors for ED visits and readmissions were assessed and further analyzed depending on the time interval between discharge from LT and readmission; within 30 days after discharge, 31 to 90 days after discharge, 91 to 180 days after discharge, 181 to 365 days after discharge, 1 year after discharge. And probabilities of ED visit and readmission over time were analyzed.

### Statistical analysis

Data were analyzed with SAS 9.3 statistical software (SAS institute Inc, Cary, NC). Cox’s proportional hazards regression model was used to compare between patients with and without history of readmission or visit to ED after LT. Multivariable analyses were performed on factors that were significantly related to readmission and visit to ED after LT with the significance level of 20% in the univariable analysis. Stepwise selection method was used in the multivariable analysis to control multicollinearity Rates of ED visits and readmissions were examined using Kaplan-Meier curves with log-rank. Univariable and multivariable analyses were performed with the same methods. P-values less than 0.05 were considered significant.

## Conclusion

More than half of the patients required ED visits or readmissions after discharge from LT, most of which occurred in the first 20 months. After discharge from LT, the 30-day and 1-year ED visit rates were 15.3% and 44.4% and the 30-day and 1-year readmission rates were 16.0% and 51.6%.
